# Supplementary figures and images for: Observational study of effects of HIV acquisition and antiretroviral treatment on biomarkers of systemic immune activation
Source: PLoS One. 2024 Jul 8;19(7):e0288895. doi: 10.1371/journal.pone.0288895 (PMC11230552; doi:10.1371/journal.pone.0288895)

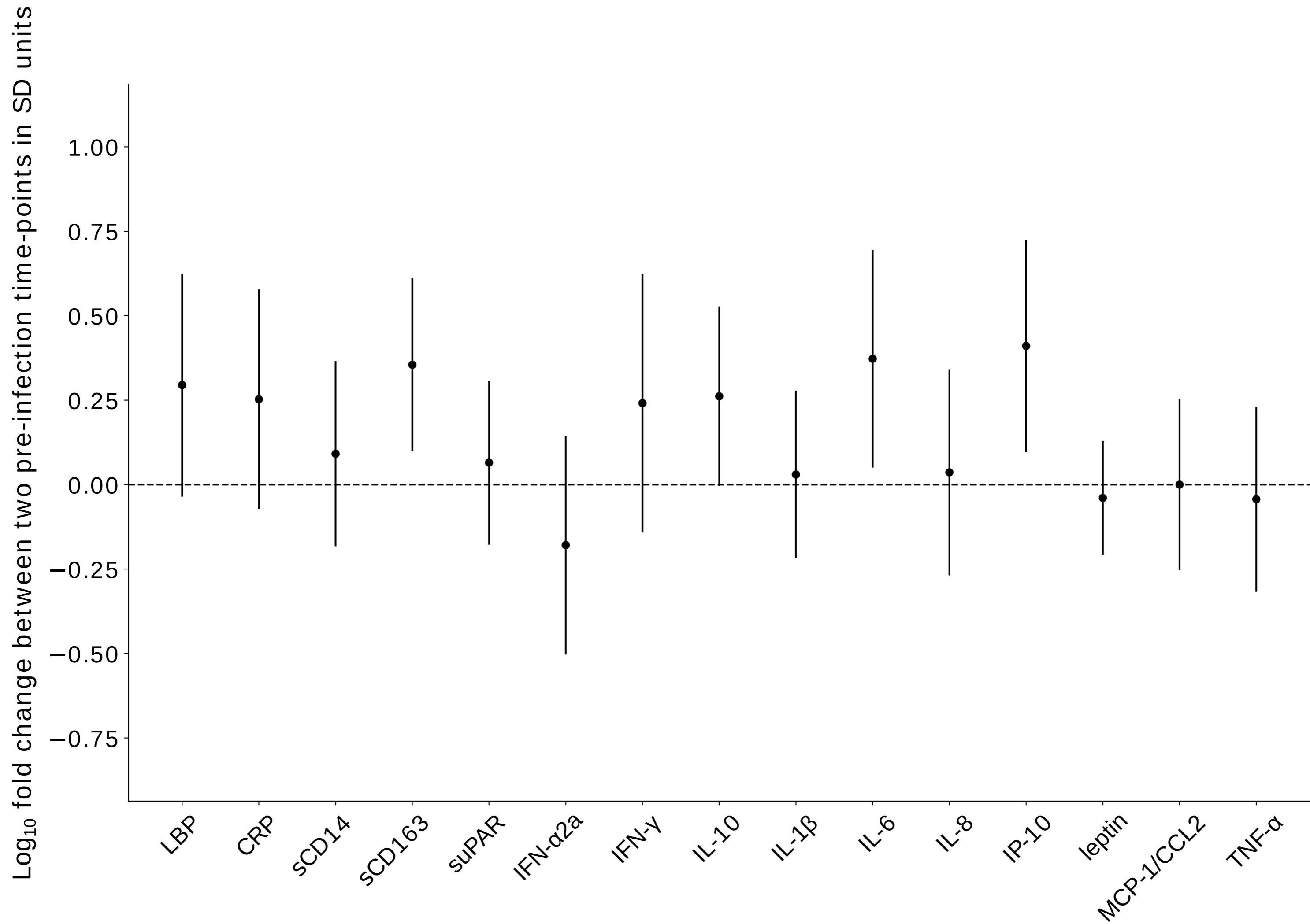

Supplement: S1 Fig — log10 biomarker levels from two specimens before HIV infection were compared for all participants (n = 50), with mean differences (dots) and 95% confidence intervals (lines) shown. The 95% confidence intervals were calculated based on a two-sided, one-sample t-test with values log10 transformed prior to analysis and differences divided by pre-infection standard deviation for each analyte. The vertical dotted line reflects no difference between two pre-HIV-infection time-points. (PDF) [file pone.0288895.s001.pdf]

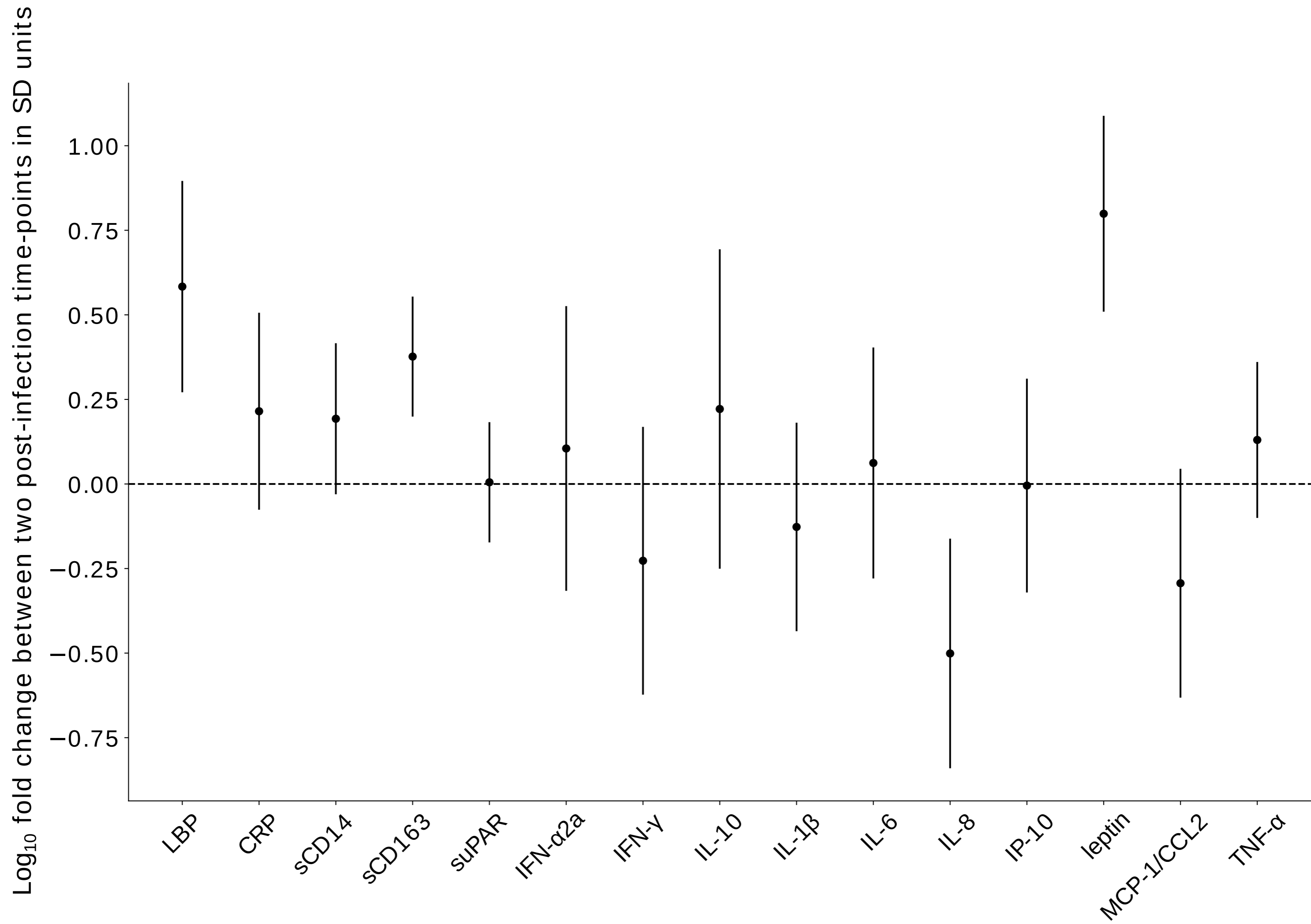

Supplement: S2 Fig — log10 biomarker levels from two specimens after HIV infection were compared for all participants (n = 50), with mean differences (dots) and 95% confidence intervals (lines) shown. The 95% confidence intervals were calculated based on a two-sided, one-sample t-test with values log10 transformed prior to analysis and differences divided by pre-infection standard deviation for each analyte. The vertical dotted line reflects no difference between two post-HIV-infection time-points. (PDF) [file pone.0288895.s002.pdf]
