# Supplementary material for: Observational study of effects of HIV acquisition and antiretroviral treatment on biomarkers of systemic immune activation
Source: PLoS One. 2024 Jul 8;19(7):e0288895. doi: 10.1371/journal.pone.0288895 (PMC11230552; doi:10.1371/journal.pone.0288895)
Supplement: S1 File — (ZIP) [file pone.0288895.s003.zip › pone.0288895_Supporting Fig legends_and _Tables 1-20-1.docx]

**Supporting information**

**S1 Fig**. **Diﬀerence between two pre-infection time-points**. log_10_ biomarker levels from two specimens before HIV infection were compared for all participants (n=50), with mean differences (dots) and 95% confidence intervals (lines) shown. The 95% confidence intervals were calculated based on a two-sided, one-sample t-test with values log_10_ transformed prior to analysis and differences divided by pre-infection standard deviation for each analyte. The vertical dotted line reflects no difference between two pre-HIV-infection time-points.

**S2 Fig**. **Diﬀerence between two post-infection time-points**. log_10_ biomarker levels from two specimens after HIV infection were compared for all participants (n=50), with mean differences (dots) and 95% confidence intervals (lines) shown. The 95% confidence intervals were calculated based on a two-sided, one-sample t-test with values log_10_ transformed prior to analysis and differences divided by pre-infection standard deviation for each analyte. The vertical dotted line reflects no difference between two post-HIV-infection time-points.

**S1 Table.** Normal ranges of biomarkers

| Biomarker | Low range | High range | Source | Biomarker | Low range | High range | Source |
| --- | --- | --- | --- | --- | --- | --- | --- |
| CD14 | 1200 ng/mL | 2600 ng/mL | Assay manufacturer: ELISAs (R&D Systems, Minneapolis, MN) | IL-10 | 0.09 pg/mL | 2.71 pg/mL | Assay manufacturer: Meso Scale Discovery (MSD, Rockville, MD) |
| CD163 | 186 ng/mL | 996 ng/mL | Assay manufacturer: ELISAs (R&D Systems, Minneapolis, MN) | IP-10 | 102 pg/mL | 676 pg/mL | Assay manufacturer: Meso Scale Discovery (MSD, Rockville, MD) |
| CRP | 0 ng/mL | 3000 ng/mL | Assay manufacturer: Meso Scale Discovery (MSD, Rockville, MD) | LBP | 2300 ng/mL | 9000 ng/mL | Assay manufacturer: Meso Scale Discovery (MSD, Rockville, MD) |
| IFN-α2a | 0 pg/mL | 2 pg/mL | Tarantino G et al. (1) | leptin | 700 pg/mL | 5300 pg/mL | Paul RF et al. (2) |
| IFN-γ | 0.46 pg/mL | 22.8 pg/mL | Assay manufacturer: Meso Scale Discovery (MSD, Rockville, MD) | MCP-1/ CCL2 | 42.3 pg/mL | 185 pg/mL | Assay manufacturer: Meso Scale Discovery (MSD, Rockville, MD) |
| IL-1b | 0.11 pg/mL | 0.94 pg/mL | Assay manufacturer: Meso Scale Discovery (MSD, Rockville, MD) | suPAR | 864 pg/mL | 3829 pg/mL | Assay manufacturer: ELISAs (R&D Systems, Minneapolis, MN) |
| IL-6 | 0.12 pg/mL | 0.99 pg/mL | Assay manufacturer: Meso Scale Discovery (MSD, Rockville, MD) | TNF-α | 0.31 pg/mL | 2.32 pg/mL | Assay manufacturer: Meso Scale Discovery (MSD, Rockville, MD) |
| IL-8 | 0.14 pg/mL | 20.4 pg/mL | Assay manufacturer: Meso Scale Discovery (MSD, Rockville, MD) |  |  |  |  |

**S2 Table.** Difference in log_10_ plasma biomarker levels between two pre-infection time-points: Visit 2 - Visit 1

| Biomarker | log_10_ FC estimate  (95% CI) | FC estimate  (95% CI) | p-value | Holm adjusted p-value |
| --- | --- | --- | --- | --- |
| CD14 | 0.009  (-0.018, 0.036) | 1.021  (0.958, 1.087) | 0.514 | 1 |
| CD163 | 0.065  (0.018, 0.112) | 1.160  (1.041, 1.293) | 0.008 | 0.121 |
| CRP | 0.141  (-0.041, 0.322) | 1.383  (0.911, 2.099) | 0.125 | 1 |
| IFN-α2a | -0.087  (-0.245, 0.070) | 0.818  (0.569, 1.174) | 0.269 | 1 |
| IFN-γ | 0.085  (-0.050, 0.220) | 1.216  (0.891, 1.661) | 0.213 | 1 |
| IL-1b | 0.016  (-0.117, 0.149) | 1.037  (0.763, 1.409) | 0.812 | 1 |
| IL-6 | 0.096  (0.013, 0.179) | 1.248  (1.030, 1.512) | 0.024 | 0.318 |
| IL-8 | 0.009  (-0.067, 0.084) | 1.020  (0.857, 1.215) | 0.821 | 1 |
| IL-10 | 0.059  (-0.001, 0.119) | 1.146  (0.998, 1.315) | 0.053 | 0.632 |
| IP-10 | 0.087  (0.020, 0.153) | 1.221  (1.048, 1.422) | 0.011 | 0.161 |
| LBP | 0.050  (-0.006, 0.106) | 1.122  (0.986, 1.276) | 0.080 | 0.882 |
| leptin | -0.021  (-0.111, 0.069) | 0.953  (0.775, 1.172) | 0.645 | 1 |
| MCP-1/ CCL2 | 0.000  (-0.031, 0.031) | 1.000  (0.931, 1.074) | 0.996 | 1 |
| suPAR | 0.013  (-0.035, 0.062) | 1.031  (0.922, 1.153) | 0.586 | 1 |
| TNF-α | -0.008  (-0.058, 0.043) | 0.983  (0.875, 1.104) | 0.764 | 1 |

Abbreviations: suPAR, soluble urokinase-type plasminogen activator receptor; sCD14 and sCD163, soluble cluster of differentiation 14 and 163; LBP, lipopolysaccharide binding protein; IL-1β, IL-6, IL-8 and IL-10, interleukin 1b, 6, 8 and 10; IFN-γ and IFN-α2a, interferon-gamma and -alpha 2a; IP-10, interferon gamma-induced protein 10; MCP-1/CCL2, monocyte chemoattractant protein-1; TNF-α, tumor necrosis factor-alpha; CRP, C-reactive protein

**S3 Table.** Difference in log_10_ plasma biomarker levels between two post-infection time-points: Visit 4 - Visit 3

| Biomarker | log_10_ FC estimate  (95% CI) | FC estimate  (95% CI) | p-value | Holm adjusted p-value |
| --- | --- | --- | --- | --- |
| CD14 | 0.019  (-0.003, 0.041) | 1.045  (0.993, 1.099) | 0.092 | 0.920 |
| CD163 | 0.069  (0.037, 0.102) | 1.172  (1.088, 1.264) | < 0.001 | 0.001 |
| CRP | 0.120  (-0.042, 0.283) | 1.319  (0.907, 1.918) | 0.144 | 1 |
| IFN-a2a | 0.051  (-0.153, 0.256) | 1.126  (0.703, 1.804) | 0.616 | 1 |
| IFN-γ | -0.080  (-0.220, 0.059) | 0.831  (0.603, 1.146) | 0.254 | 1 |
| IL-1b | -0.068  (-0.233, 0.097) | 0.855  (0.585, 1.249) | 0.410 | 1 |
| IL-6 | 0.016  (-0.072, 0.104) | 1.038  (0.848, 1.271) | 0.712 | 1 |
| IL-8 | -0.124  (-0.208, -0.040) | 0.752  (0.619, 0.913) | 0.005 | 0.058 |
| IL-10 | 0.050  (-0.057, 0.156) | 1.121  (0.878, 1.431) | 0.353 | 1 |
| IP-10 | -0.001  (-0.068, 0.066) | 0.997  (0.855, 1.163) | 0.970 | 1 |
| LBP | 0.099  (0.046, 0.152) | 1.257  (1.112, 1.420) | < 0.001 | 0.006 |
| leptin | 0.425  (0.271, 0.579) | 2.659  (1.865, 3.792) | < 0.001 | < 0.001 |
| MCP-1/ CCL2 | -0.036  (-0.078, 0.005) | 0.920  (0.836, 1.012) | 0.083 | 0.916 |
| suPAR | 0.001  (-0.034, 0.037) | 1.003  (0.925, 1.088) | 0.939 | 1 |
| TNF-a | 0.024  (-0.018, 0.067) | 1.058  (0.958, 1.168) | 0.258 | 1 |

Abbreviations: suPAR, soluble urokinase-type plasminogen activator receptor; sCD14 and sCD163, soluble cluster of differentiation 14 and 163; LBP, lipopolysaccharide binding protein; IL-1β, IL-6, IL-8 and IL-10, interleukin 1b, 6, 8 and 10; IFN-γ and IFN-α2a, interferon-gamma and -alpha 2a; IP-10, interferon gamma-induced protein 10; MCP-1/CCL2, monocyte chemoattractant protein-1; TNF-α, tumor necrosis factor-alpha; CRP, C-reactive protein

**Model 1 (S4, S8, S11 and S12 Tables )** evaluates post- vs pre-infection biomarker values, ignoring timing of ART-initiation: **Biomarker** level ~ Participant + After_infection [0 pre-infection or 1 post-infection]

For each biomarker we present estimates of the difference in plasma levels due to HIV-1 infection but following effective treatment, and 95% confidence intervals, in addition to unadjusted p-values. All biomarker values were log10 transformed prior to analysis. When this p-value is small, there is evidence for a change in biomarker levels irrespective of the timing of ART-initiation. Estimates and confidence intervals are expressed as a difference in means on the log10 scale, and can be interpreted as a log10 fold-change of geometric means between the biomarker values measured in the two conditions. We report results for both the ‘as-treated’ assignment (S4 and S8 Tables) and ‘per-protocol’ (S11 and S12 Tables).We performed analysis using all timepoints (S4, S11 Tables) for the whole set of biomarkers. For the unstable biomarkers we also performed analysis using just visit 2 and visit 4 (S8 and S12 Tables). P-values were Holm adjusted within each set; S4 and S11 Tables over 15 biomarkers, S8 and S12 Tables over 6 biomarkers.

**S4 Table.** Difference in log_10_ plasma biomarker levels from pre- to post-infection/ART-suppression (“as-treated” analysis, regardless of timing of ART-initiation)

| Biomarker | log10 FC estimate  (95% CI) | FC estimate  (95% CI) | p-value | Holm adjusted p-value |
| --- | --- | --- | --- | --- |
| CD14 | 0.000  (-0.018, 0.018) | 1 .000 (0.960, 1.042) | 0.991 | 1 |
| CD163 | -0.001  (-0.032, 0.030) | 0.998  (0.930, 1.071) | 0.955 | 1 |
| CRP | 0.210  (0.091, 0.330) | 1.623  (1.232, 2.138) | 0.001 | 0.008 |
| IFN-α2a | -0.059  (-0.194, 0.075) | 0.872  (0.640, 1.189) | 0.384 | 1 |
| IFN-γ | -0.049  (-0.142, 0.044) | 0.893  (0.721, 1.107) | 0.299 | 1 |
| IL-1b | 0.001  (-0.114, 0.117) | 1.003  (0.770, 1.308) | 0.981 | 1 |
| IL-6 | -0.008  (-0.074, 0.057) | 0.981  (0.843, 1.141) | 0.803 | 1 |
| IL-8 | -0.036  (-0.090, 0.018) | 0.921  (0.813, 1.043) | 0.192 | 1 |
| IL-10 | 0.002  (-0.058, 0.062) | 1.005  (0.875, 1.154) | 0.944 | 1 |
| IP-10 | 0.138  (0.094, 0.181) | 1.373  (1.241, 1.519) | < 0.001 | < 0.001 |
| LBP | -0.056  (-0.095, -0.016) | 0.879  (0.803, 0.963) | 0.006 | 0.066 |
| leptin | -0.281  (-0.392, -0.170) | 0.523  (0.406, 0.675) | < 0.001 | < 0.001 |
| MCP-1/ CCL2 | 0.058  (0.031, 0.085) | 1.143  (1.074, 1.216) | < 0.001 | 0.001 |
| suPAR | -0.004  (-0.036, 0.028) | 0.991  (0.921, 1.067) | 0.815 | 1 |
| TNF-α | 0.039  (0.004, 0.074) | 1.094  (1.010, 1.185) | 0.028 | 0.276 |

Abbreviations: suPAR, soluble urokinase-type plasminogen activator receptor; sCD14 and sCD163, soluble cluster of differentiation 14 and 163; LBP, lipopolysaccharide binding protein; IL-1β, IL-6, IL-8 and IL-10, interleukin 1b, 6, 8 and 10; IFN-γ and IFN-α2a, interferon-gamma and -alpha 2a; IP-10, interferon gamma-induced protein 10; MCP-1/CCL2, monocyte chemoattractant protein-1; TNF-α, tumor necrosis factor-alpha; CRP, C-reactive protein

**Model 2 (S5, S6, S7, S9-S18 Tables)** evaluates the impact of very early ART initiation on the change in biomarkers post- vs pre-infection: Biomarker level ~ Participant + After_infection [0 pre-infection or 1 post-infection] + After_infection:ART_start

The biomarker measurements at pre-ART time points are structurally independent of the ART start group by study design, so we drop the 'ART_ start’ term to ensure identifiability. All biomarker values were log10 transformed prior to analysis. In Supplementary Table 5 we report unadjusted F-test p-values for the comparison of model 1 and model 2. When this p-value is significant, the difference between the post-infection and pre-infection biomarker values differ by timing of ART-initiation group. In S10-S18 Tables we report 95% confidence intervals and unadjusted p-values for the term reflecting the post- vs pre- infection differences within an immediate (and respectively, deferred) ART-initiation group. When this p-value is significant, there is evidence for a change in biomarker levels in participants who initiated ART in the immediate-ART timeframe (and resp. deferred). Estimates and confidence intervals are expressed as a difference in means on the log_10_ scale, and can be interpreted as a log_10_ fold-change of geometric means between the biomarker values measured in the two conditions. We report results for both the ‘as-treated’ assignment (S5, S6, S9, S10, S11 Tables) and ‘per-protocol’ (S5, S6, S14, S16, S18 Tables). Analysis was performed using all timepoints (S5, S6, S13, S15, S17 Tables) for the whole set of biomarkers. For the unstable biomarkers also analysis using just visit 2 and visit 4 was performed (S9, S10, S11, S14, S16, S18 Tables). P-values were Holm adjusted within each set; S5, S6, S7, S15, S17 over 15 biomarkers, S9, S10, S11, S14, S16, S18 Tables over 6 biomarkers.

**S5 Table.** Difference in change in log_10_ plasma biomarker levels from pre- to post-infection/ART-suppression between immediate vs. deferred ART-initiation (“as-treated” analysis)

| Biomarker | p-value | Holm adjusted p-value | biomarker | p-value | Holm adjusted p-value |
| --- | --- | --- | --- | --- | --- |
| CD14 | 0.711 | 1 | **IL-10** | 0.602 | 1 |
| CD163 | 0.411 | 1 | **IP-10** | 0.890 | 1 |
| CRP | 0.040 | 0.565 | **LBP** | 0.290 | 1 |
| IFN-α2a | 0.016 | 0.241 | **leptin** | 0.626 | 1 |
| IFN-γ | 0.287 | 1 | **MCP-1/CCL2** | 0.395 | 1 |
| IL-1b | 0.743 | 1 | **suPAR** | 0.658 | 1 |
| IL-6 | 0.217 | 1 | **TNF-α** | 0.926 | 1 |
| IL-8 | 0.692 | 1 |  |  |  |

Abbreviations: suPAR, soluble urokinase-type plasminogen activator receptor; sCD14 and sCD163, soluble cluster of differentiation 14 and 163; LBP, lipopolysaccharide binding protein; IL-1β, IL-6, IL-8 and IL-10, interleukin 1b, 6, 8 and 10; IFN-γ and IFN-α2a, interferon-gamma and -alpha 2a; IP-10, interferon gamma-induced protein 10; MCP-1/CCL2, monocyte chemoattractant protein-1; TNF-α, tumor necrosis factor-alpha; CRP, C-reactive protein

**S6 Table.** Difference in log_10_ plasma biomarker levels from pre- to post-infection/ART-suppression among deferred-ART group (“as-treated” analysis)

| Biomarker | log10 FC estimate  (95% CI) | FC estimate  (95% CI) | p-value | Holm adjusted p-value |
| --- | --- | --- | --- | --- |
| CD14 | 0.003  (-0.021, 0.027) | 1.007  (0.953, 1.065) | 0.798 | 1 |
| CD163 | 0.011  (-0.031, 0.052) | 1.025  (0.932, 1.127) | 0.611 | 1 |
| CRP | 0.322  (0.163, 0.481) | 2.097  (1.454, 3.025) | < 0.001 | 0.001 |
| IFN-α2a | -0.205  (-0.383, -0.028) | 0.623  (0.414, 0.937) | 0.024 | 0.259 |
| IFN-γ | -0.004  (-0.129, 0.121) | 0.991  (0.743, 1.322) | 0.952 | 1 |
| IL-1b | 0.019  (-0.137, 0.174) | 1.044  (0.730, 1.493) | 0.813 | 1 |
| IL-6 | 0.029  (-0.060, 0.117) | 1.068  (0.872, 1.309) | 0.522 | 1 |
| IL-8 | -0.026  (-0.099, 0.047) | 0.942  (0.796, 1.114) | 0.481 | 1 |
| IL-10 | 0.016  (-0.065, 0.097) | 1.039  (0.862, 1.252) | 0.689 | 1 |
| IP-10 | 0.140  (0.081, 0.200) | 1.381  (1.205, 1.583) | < 0.001 | < 0.001 |
| LBP | -0.037  (-0.090, 0.016) | 0.919  (0.813, 1.038) | 0.173 | 1 |
| leptin | -0.306  (-0.455, -0.157) | 0.495  (0.351, 0.697) | < 0.001 | 0.001 |
| MCP-1/ CCL2 | 0.047  (0.011, 0.084) | 1.115  (1.026, 1.213) | 0.011 | 0.133 |
| suPAR | -0.010  (-0.053, 0.033) | 0.977  (0.885, 1.078) | 0.639 | 1 |
| TNF-α | 0.038  (-0.009, 0.084) | 1.090  (0.979, 1.214) | 0.115 | 1 |

Abbreviations: suPAR, soluble urokinase-type plasminogen activator receptor; sCD14 and sCD163, soluble cluster of differentiation 14 and 163; LBP, lipopolysaccharide binding protein; IL-1β, IL-6, IL-8 and IL-10, interleukin 1b, 6, 8 and 10; IFN-γ and IFN-α2a, interferon-gamma and -alpha 2a; IP-10, interferon gamma-induced protein 10; MCP-1/CCL2, monocyte chemoattractant protein-1; TNF-α, tumor necrosis factor-alpha; CRP, C-reactive protein

**S7 Table.** Difference in log_10_ plasma biomarker levels from pre- to post-infection/ART-suppression among immediate-ART group (“as-treated” analysis)

| Biomarker | log10 FC estimate  (95% CI) | FC estimate  (95% CI) | p-value | Holm adjusted p-value |
| --- | --- | --- | --- | --- |
| CD14 | -0.004  (-0.031, 0.023) | 0.992  (0.932, 1.055) | 0.788 | 1 |
| CD163 | -0.015  (-0.061, 0.031) | 0.966  (0.869, 1.074) | 0.516 | 1 |
| CRP | 0.073  (-0.104, 0.250) | 1.182  (0.786, 1.777) | 0.419 | 1 |
| IFN-α2a | 0.122  (-0.076, 0.319) | 1.324  (0.840, 2.086) | 0.225 | 1 |
| IFN-γ | -0.105  (-0.244, 0.034) | 0.785  (0.570, 1.082) | 0.138 | 1 |
| IL-1b | -0.020  (-0.193, 0.153) | 0.955  (0.642, 1.422) | 0.820 | 1 |
| IL-6 | -0.054  (-0.152, 0.044) | 0.883  (0.704, 1.107) | 0.278 | 1 |
| IL-8 | -0.048  (-0.129, 0.033) | 0.895  (0.742, 1.080) | 0.245 | 1 |
| IL-10 | -0.016  (-0.106, 0.075) | 0.965  (0.784, 1.187) | 0.733 | 1 |
| IP-10 | 0.134  (0.068, 0.200) | 1.362  (1.170, 1.585) | < 0.001 | 0.001 |
| LBP | -0.080  (-0.139, -0.020) | 0.833  (0.727, 0.954) | 0.009 | 0.106 |
| leptin | -0.251  (-0.417, -0.085) | 0.562  (0.383, 0.823) | 0.003 | 0.044 |
| MCP-1/ CCL2 | 0.071  (0.030, 0.111) | 1.177  (1.072, 1.293) | 0.001 | 0.010 |
| suPAR | 0.004  (-0.044, 0.052) | 1.010  (0.905, 1.127) | 0.862 | 1 |
| TNF-α | 0.041  (-0.011, 0.093) | 1.099  (0.975, 1.239) | 0.123 | 1 |

Abbreviations: suPAR, soluble urokinase-type plasminogen activator receptor; sCD14 and sCD163, soluble cluster of differentiation 14 and 163; LBP, lipopolysaccharide binding protein; IL-1β, IL-6, IL-8 and IL-10, interleukin 1b, 6, 8 and 10; IFN-γ and IFN-α2a, interferon-gamma and -alpha 2a; IP-10, interferon gamma-induced protein 10; MCP-1/CCL2, monocyte chemoattractant protein-1; TNF-α, tumor necrosis factor-alpha; CRP, C-reactive protein

**S8 Table**. Difference in log_10_ plasma biomarker levels from pre- to post-infection/ART-suppression (“as-treated” analysis, regardless of timing of ART-initiation) for the unstable biomarkers: Visit 4 - Visit 2

| Biomarker | log_10_ FC estimate  (95% CI) | FC estimate  (95% CI) | p-value | Holm adjusted p-value |
| --- | --- | --- | --- | --- |
| CD163 | 0.008  (-0.038, 0.053) | 1.018  (0.917, 1.131) | 0.728 | 1 |
| IL-6 | -0.047  (-0.148, 0.054) | 0.898  (0.712, 1.132) | 0.355 | 1 |
| IL-8 | -0.108  (-0.182, -0.034) | 0.780  (0.658, 0.925) | 0.005 | 0.026 |
| IP-10 | 0.103  (0.037, 0.169) | 1.267  (1.088, 1.474) | 0.003 | 0.018 |
| LBP | -0.026  (-0.078, 0.026) | 0.942  (0.835, 1.063) | 0.323 | 1 |
| leptin | -0.049  (-0.191, 0.094) | 0.894  (0.644, 1.241) | 0.495 | 1 |

Abbreviations: sCD163, soluble cluster of differentiation 163; LBP, lipopolysaccharide binding protein; IL-6, and IL-8, interleukin 6, and 8; IP-10, interferon gamma-induced protein 10

**S9 Table**. Difference in change in log_10_ plasma biomarker levels from pre- to post-infection/ART-suppression between immediate vs. deferred ART-initiation (“as-treated” analysis) for the unstable biomarkers: Visit 4 - Visit 2

| Biomarker | p-value | Holm adjusted p-value | Biomarker | p-value | Holm adjusted p-value |
| --- | --- | --- | --- | --- | --- |
| CD163 | 0.408 | 1 | **IP-10** | 0.321 | 1 |
| IL-6 | 0.224 | 1 | **LBP** | 0.625 | 1 |
| IL-8 | 0.781 | 1 | **leptin** | 0.930 | 1 |

Abbreviations: sCD163, soluble cluster of differentiation 163; LBP, lipopolysaccharide binding protein; IL-6, and IL-8, interleukin 6, and 8; IP-10, interferon gamma-induced protein 10

**S10 Table**. Difference in log_10_ plasma biomarker levels from pre- to post-infection/ART-suppression among deferred-ART group (“as-treated” analysis) for the unstable biomarkers: Visit 4 - Visit 2

| Biomarker | log10 FC estimate  (95% CI) | FC estimate  (95% CI) | p-value | Holm adjusted p-value |
| --- | --- | --- | --- | --- |
| CD163 | 0.025  (-0.037, 0.086) | 1.059  (0.919, 1.220) | 0.418 | 1 |
| IL-6 | 0.008  (-0.126, 0.143) | 1.019  (0.747, 1.390) | 0.901 | 1 |
| IL-8 | -0.098  (-0.199, 0.002) | 0.797  (0.632, 1.005) | 0.055 | 0.328 |
| IP-10 | 0.073  (-0.016, 0.162) | 1.183  (0.964, 1.451) | 0.104 | 0.522 |
| LBP | -0.014  (-0.085, 0.057) | 0.968  (0.822, 1.139) | 0.686 | 1 |
| leptin | -0.043  (-0.237, 0.151) | 0.905  (0.579, 1.415) | 0.657 | 1 |

Abbreviations: sCD163, soluble cluster of differentiation 163; LBP, lipopolysaccharide binding protein; IL-6, and IL-8, interleukin 6, and 8; IP-10, interferon gamma-induced protein 10

**S11 Table.** Difference in log_10_ plasma biomarker levels from pre- to post-infection/ART-suppression among immediate-ART group (“as-treated” analysis) for the unstable biomarkers: Visit 4 - Visit 2.

| Biomarker | log10 FC estimate  (95% CI) | FC estimate  (95% CI) | p-value | Holm adjusted p-value |
| --- | --- | --- | --- | --- |
| CD163 | -0.013  (-0.082, 0.055) | 0.970  (0.829, 1.136) | 0.700 | 1 |
| IL-6 | -0.115  (-0.265, 0.035) | 0.767  (0.543, 1.084) | 0.130 | 0.518 |
| IL-8 | -0.119  (-0.231, -0.008) | 0.760  (0.587, 0.983) | 0.037 | 0.185 |
| IP-10 | 0.139  (0.040, 0.238) | 1.378  (1.098, 1.730) | 0.007 | 0.041 |
| LBP | -0.040  (-0.119, 0.039) | 0.911  (0.760, 1.093) | 0.309 | 0.928 |
| leptin | -0.056  (-0.272, 0.160) | 0.879  (0.535, 1.446) | 0.605 | 1 |

Abbreviations: sCD163, soluble cluster of differentiation 163; LBP, lipopolysaccharide binding protein; IL-6, and IL-8, interleukin 6, and 8; IP-10, interferon gamma-induced protein 10

**S12 Table.** Difference in log_10_ plasma biomarker levels from pre- to post-infection/ART-suppression (“per-protocol” analysis, regardless of timing of ART-initiation)

| Biomarker | log10 FC estimate  (95% CI) | FC estimate  (95% CI) | p-value | Holm adjusted p-value |
| --- | --- | --- | --- | --- |
| CD14 | 0.002  (-0.016, 0.020) | 1.004 (0.963, 1.047) | 0.839 | 1 |
| CD163 | -0.006  (-0.038, 0.026) | 0.987 (0.916, 1.063) | 0.723 | 1 |
| CRP | 0.206  (0.085, 0.327) | 1.608 (1.217, 2.124) | 0.001 | 0.011 |
| IFN-α2a | -0.029 (-0.164, 0.105) | 0.935 (0.686, 1.274) | 0.668 | 1 |
| IFN-γ | -0.044  (-0.138, 0.051) | 0.905 (0.728, 1.125) | 0.365 | 1 |
| IL-1b | 0.042 (-0.075, 0.159) | 1.101 (0.841, 1.442) | 0.481 | 1 |
| IL-6 | -0.008  (-0.073, 0.057) | 0.983 (0.846, 1.142) | 0.817 | 1 |
| IL-8 | -0.021  (-0.078, 0.035) | 0.952 (0.836, 1.083) | 0.450 | 1 |
| IL-10 | 0.018  (-0.044, 0.080) | 1.043 (0.903, 1.204) | 0.565 | 1 |
| IP-10 | 0.153  (0.106, 0.200) | 1.422 (1.276, 1.585) | < 0.001 | < 0.001 |
| LBP | -0.057  (-0.097, -0.017) | 0.877 (0.800, 0.962) | 0.006 | 0.059 |
| leptin | -0.279  (-0.384, -0.175) | 0.526 (0.413, 0.669) | < 0.001 | < 0.001 |
| MCP-1/ CCL2 | 0.063  (0.036, 0.089) | 1.155 (1.088, 1.227) | < 0.001 | < 0.001 |
| suPAR | -0.015  (-0.05, 0.020) | 0.966 (0.891, 1.048) | 0.406 | 1 |
| TNF-α | 0.050  (0.015, 0.086) | 1.123 (1.035, 1.218) | 0.005 | 0.059 |

Abbreviations: suPAR, soluble urokinase-type plasminogen activator receptor; sCD14 and sCD163, soluble cluster of differentiation 14 and 163; LBP, lipopolysaccharide binding protein; IL-1β, IL-6, IL-8 and IL-10, interleukin 1b, 6, 8 and 10; IFN-γ and IFN-α2a, interferon-gamma and -alpha 2a; IP-10, interferon gamma-induced protein 10; MCP-1/CCL2, monocyte chemoattractant protein-1; TNF-α, tumor necrosis factor-alpha; CRP, C-reactive protein

**S13 Table. Difference in log_10_ plasma biomarker levels from pre- to post-infection/ART-suppression (“per-protocol” analysis, regardless of timing of ART-initiation) for the unstable biomarkers: Visit 4 - Visit 2**

| Biomarker | log10 FC estimate  (95% CI) | FC estimate  (95% CI) | p-value | Holm adjusted p-value |
| --- | --- | --- | --- | --- |
| CD163 | -0.004  (-0.054, 0.047) | 0.992  (0.883, 1.114) | 0.888 | 1 |
| IL-6 | -0.048  (-0.151, 0.055) | 0.896  (0.707, 1.136) | 0.358 | 1 |
| IL-8 | -0.088  (-0.168, -0.007) | 0.817  (0.679, 0.983) | 0.033 | 0.164 |
| IP-10 | 0.109  (0.040, 0.178) | 1.285  (1.096, 1.507) | 0.003 | 0.016 |
| LBP | -0.032  (-0.085, 0.021) | 0.929  (0.822, 1.050) | 0.232 | 0.929 |
| leptin | -0.056  (-0.192, 0.079) | 0.878  (0.643, 1.200) | 0.407 | 1 |

Abbreviations: sCD163, soluble cluster of differentiation 163; LBP, lipopolysaccharide binding protein; IL-6, and IL-8, interleukin 6, and 8; IP-10, interferon gamma-induced protein 10

**S14 Table.** Difference in change in log_10_ plasma biomarker levels from pre- to post-infection/ART-suppression between immediate vs. deferred ART-initiation (“per-protocol” analysis)

| Biomarker | p-value | Holm adjusted p-value | Biomarker | p-value | Holm adjusted p-value |
| --- | --- | --- | --- | --- | --- |
| CD14 | 0.833 | 1 | **IL-10** | 0.64 | 1 |
| CD163 | 0.675 | 1 | **IP-10** | 0.326 | 1 |
| CRP | 0.027 | 0.406 | **LBP** | 0.091 | 1 |
| IFN-α2a | 0.044 | 0.612 | **leptin** | 0.551 | 1 |
| IFN-γ | 0.223 | 1 | **MCP-1/CCL2** | 0.739 | 1 |
| IL-1b | 0.999 | 1 | **suPAR** | 0.282 | 1 |
| IL-6 | 0.133 | 1 | **TNF-α** | 0.392 | 1 |
| IL-8 | 0.744 | 1 |  |  |  |

Abbreviations: suPAR, soluble urokinase-type plasminogen activator receptor; sCD14 and sCD163, soluble cluster of differentiation 14 and 163; LBP, lipopolysaccharide binding protein; IL-1β, IL-6, IL-8 and IL-10, interleukin 1b, 6, 8 and 10; IFN-γ and IFN-α2a, interferon-gamma and -alpha 2a; IP-10, interferon gamma-induced protein 10; MCP-1/CCL2, monocyte chemoattractant protein-1; TNF-α, tumor necrosis factor-alpha; CRP, C-reactive protein

**S15 Table.** Difference in change in log_10_ plasma biomarker levels from pre- to post-infection/ART-suppression between immediate vs. deferred ART-initiation (“per-protocol” analysis) for the unstable biomarkers: Visit 4 - Visit 2

| Biomarker | p-value | Holm adjusted p-value | Biomarker | p-value | Holm adjusted p-value |
| --- | --- | --- | --- | --- | --- |
| CD163 | 0.580 | 1 | **IP-10** | 0.500 | 1 |
| IL-6 | 0.163 | 0.975 | **LBP** | 0.355 | 1 |
| IL-8 | 0.643 | 1 | **leptin** | 0.768 | 1 |

Abbreviations: sCD163, soluble cluster of differentiation 163; LBP, lipopolysaccharide binding protein; IL-6, and IL-8, interleukin 6, and 8; IP-10, interferon gamma-induced protein 10

**S16 Table** . Difference in log_10_ plasma biomarker levels from pre- to post-infection/ART-suppression among deferred-ART group (“per-protocol” analysis)

| Biomarker | log10 FC estimate  (95% CI) | FC estimate  (95% CI) | p-value | Holm adjusted p-value |
| --- | --- | --- | --- | --- |
| CD14 | 0.000  (-0.023, 0.023) | 1.001  (0.949, 1.055) | 0.976 | 1 |
| CD163 | -0.011  (-0.052, 0.030) | 0.975  (0.887, 1.071) | 0.591 | 1 |
| CRP | 0.312  (0.160, 0.463) | 2.050  (1.446, 2.906) | <0.001 | 0.001 |
| IFN-α2a | -0.137  (-0.306, 0.033) | 0.730  (0.495, 1.078) | 0.113 | 1 |
| IFN-γ | 0.002  (-0.118, 0.122) | 1.005  (0.763, 1.325) | 0.970 | 1 |
| IL-1b | 0.042  (-0.107, 0.191) | 1.101  (0.781, 1.552) | 0.580 | 1 |
| IL-6 | 0.031  (-0.051, 0.114) | 1.074  (0.889, 1.299) | 0.456 | 1 |
| IL-8 | -0.014  (-0.086, 0.057) | 0.968  (0.821, 1.141) | 0.695 | 1 |
| IL-10 | 0.030  (-0.050, 0.109) | 1.071  (0.892, 1.286) | 0.459 | 1 |
| IP-10 | 0.171  (0.111, 0.231) | 1.484  (1.293, 1.703) | < 0.001 | < 0.001 |
| LBP | -0.030  (-0.080, 0.021) | 0.933  (0.831, 1.048) | 0.243 | 1 |
| leptin | -0.304  (-0.437, -0.171) | 0.497  (0.366, 0.675) | < 0.001 | < 0.001 |
| MCP-1/ CCL2 | 0.059  (0.026, 0.093) | 1.146  (1.061, 1.238) | 0.001 | 0.007 |
| suPAR | -0.030  (-0.074, 0.015) | 0.934  (0.843, 1.035) | 0.188 | 1 |
| TNF-α | 0.062  (0.018, 0.107) | 1.154  (1.041, 1.208) | 0.007 | 0.074 |

Abbreviations: suPAR, soluble urokinase-type plasminogen activator receptor; sCD14 and sCD163, soluble cluster of differentiation 14 and 163; LBP, lipopolysaccharide binding protein; IL-1β, IL-6, IL-8 and IL-10, interleukin 1b, 6, 8 and 10; IFN-γ and IFN-α2a, interferon-gamma and -alpha 2a; IP-10, interferon gamma-induced protein 10; MCP-1/CCL2, monocyte chemoattractant protein-1; TNF-α, tumor necrosis factor-alpha; CRP, C-reactive protein

**S17 Table**. Difference in log_10_ plasma biomarker levels from pre- to post-infection/ART-suppression among deferred-ART group (“per-protocol” analysis) for the unstable biomarkers: Visit 4 - Visit 2

| Biomarker | log10 FC estimate  (95% CI) | FC estimate  (95% CI) | p-value | Holm adjusted p-value |
| --- | --- | --- | --- | --- |
| CD163 | -0.015  (-0.079, 0.050) | 0.967  (0.834, 1.122) | 0.652 | 1 |
| IL-6 | 0.009  (-0.121, 0.138) | 1.020  (0.757, 1.375) | 0.893 | 1 |
| IL-8 | -0.073  (-0.176, 0.030) | 0.845  (0.667, 1.071) | 0.160 | 0.798 |
| IP-10 | 0.091  (0.002, 0.179) | 1.232  (1.005, 1.510) | 0.044 | 0.266 |
| LBP | -0.013  (-0.080, 0.055) | 0.971  (0.831, 1.135) | 0.709 | 1 |
| leptin | -0.041  (-0.214, 0.133) | 0.911  (0.610, 1.359) | 0.641 | 1 |

Abbreviations: sCD163, soluble cluster of differentiation 163; LBP, lipopolysaccharide binding protein; IL-6, and IL-8, interleukin 6, and 8; IP-10, interferon gamma-induced protein 10

**S18 Table.** Difference in log_10_ plasma biomarker levels from pre- to post-infection/ART-suppression among immediate-ART group (“per-protocol” analysis)

| Biomarker | log10 FC estimate  (95% CI) | FC estimate  (95% CI) | p-value | Holm adjusted p-value |
| --- | --- | --- | --- | --- |
| CD14 | 0.004  (-0.025, 0.034) | 1.010  (0.944, 1.081) | 0.771 | 1 |
| CD163 | 0.003  (-0.049, 0.055) | 1.007  (0.892, 1.136) | 0.911 | 1 |
| CRP | 0.034  (-0.159, 0.228) | 1.082  (0.693, 1.689) | 0.728 | 1 |
| IFN-α2a | 0.146  (-0.070, 0.362) | 1.399  (0.851, 2.301) | 0.184 | 1 |
| IFN-γ | -0.118  (-0.271, 0.035) | 0.762  (0.535, 1.084) | 0.129 | 1 |
| IL-1b | 0.042  (-0.148, 0.232) | 1.101  (0.710, 1.708) | 0.664 | 1 |
| IL-6 | -0.071  (-0.176, 0.034) | 0.849  (0.667, 1.082) | 0.185 | 1 |
| IL-8 | -0.033  (-0.125, 0.058) | 0.926  (0.751, 1.142) | 0.471 | 1 |
| IL-10 | -0.001  (-0.102, 0.101) | 0.998  (0.791, 1.261) | 0.988 | 1 |
| IP-10 | 0.123  (0.046, 0.199) | 1.327  (1.113, 1.583) | 0.002 | 0.027 |
| LBP | -0.101  (-0.165, -0.036) | 0.793  (0.684, 0.920) | 0.002 | 0.032 |
| leptin | -0.239  (-0.409, -0.069) | 0.577  (0.390, 0.854) | 0.006 | 0.075 |
| MCP-1/ CCL2 | 0.068  (0.026, 0.111) | 1.170  (1.061, 1.291) | 0.002 | 0.027 |
| suPAR | 0.010  (-0.047, 0.067) | 1.023  (0.897, 1.166) | 0.737 | 1 |
| TNF-α | 0.031  (-0.026, 0.088) | 1.073  (0.941, 1.224 | 0.290 | 1 |

Abbreviations: suPAR, soluble urokinase-type plasminogen activator receptor; sCD14 and sCD163, soluble cluster of differentiation 14 and 163; LBP, lipopolysaccharide binding protein; IL-1β, IL-6, IL-8 and IL-10, interleukin 1b, 6, 8 and 10; IFN-γ and IFN-α2a, interferon-gamma and -alpha 2a; IP-10, interferon gamma-induced protein 10; MCP-1/CCL2, monocyte chemoattractant protein-1; TNF-α, tumor necrosis factor-alpha; CRP, C-reactive protein

**S19 Table**. Difference in log_10_ plasma biomarker levels from pre- to post-infection/ART-suppression among immediate-ART group (“per-protocol” analysis) for the unstable biomarkers: Visit 4 - Visit 2

| Biomarker | log10 FC estimate  (95% CI) | FC estimate  (95% CI) | p-value | Holm adjusted p-value |
| --- | --- | --- | --- | --- |
| CD163 | 0.014  (-0.068, 0.097) | 1.034  (0.855, 1.250) | 0.726 | 0.921 |
| IL-6 | -0.140  (-0.305, 0.026) | 0.725  (0.495, 1.062) | 0.097 | 0.468 |
| IL-8 | -0.112  (-0.243, 0.020) | 0.773  (0.571, 1.046) | 0.094 | 0.468 |
| IP-10 | 0.139  (0.026, 0.252) | 1.377  (1.062, 1.786) | 0.017 | 0.100 |
| LBP | -0.064  (-0.150, 0.023) | 0.863  (0.707, 1.054) | 0.145 | 0.468 |
| leptin | -0.082  (-0.304, 0.140) | 0.828  (0.496, 1.380) | 0.461 | 0.921 |

Abbreviations: sCD163, soluble cluster of differentiation 163; LBP, lipopolysaccharide binding protein; IL-6, and IL-8, interleukin 6, and 8; IP-10, interferon gamma-induced protein 10

REFERENCES

1. Tarantino G, Costantini S, Citro V, Conforti P, Capone F, Sorice A, et al. Interferon-alpha 2 but not Interferon-gamma serum levels are associated with intramuscular fat in obese patients with nonalcoholic fatty liver disease. Journal of translational medicine. 2019;17(1):1-14.
2. Paul RF, Hassan M, Nazar HS, Gillani S, Afzal N, Qayyum I. Effect of body mass index on serum leptin levels. J Ayub Med Coll Abbottabad. 2011;23(3):40-3.
