# Supplementary material for: Observational study of effects of HIV acquisition and antiretroviral treatment on biomarkers of systemic immune activation
Source: PLoS One. 2024 Jul 8;19(7):e0288895. doi: 10.1371/journal.pone.0288895 (PMC11230552; doi:10.1371/journal.pone.0288895)
Supplement: Attachment — Submitted filename: Response_reviewers_PLoS_One-19nov23.pdf [file pone.0288895.s005.pdf]

Editor’s and Reviewers’ comments and authors’ responses

1. Please ensure that your manuscript meets PLOS ONE's style requirements, including those for file naming. The PLOS ONE style templates can be found at

https://journals.plos.org/plosone/s/file?id=wjVg/PLOSOne_formatting_sample_main_body.pdf

and

https://journals.plos.org/plosone/s/file?id=ba62/PLOSOne_formatting_sample_title_authors_affiliations.pdf

Response: In revising the manuscript we have followed the style requirements listed at the links above.

2. Thank you for stating the following financial disclosure:

“The project was funded by NIH R01 DA040532 (ACD). Additional support came from the Molecular Profiling and Computational Biology Core of the University of Washington and Fred Hutch Center for AIDS Research (award number P30 AI027757), from the Laboratory Core of International Maternal Pediatric Adolescent AIDS Clinical Trials Network (IMPAACT) UM1 AI106716 (Subaward: LMF). This work was also supported by the National Center For Advancing Translational Sciences of the National Institutes of Health under Award Number KL2 TR002317 (GG). The content is solely the responsibility of the authors and does not necessarily represent the official views of the National Institutes of Health.”

Please state what role the funders took in the study. If the funders had no role, please state: "The funders had no role in study design, data collection and analysis, decision to publish, or preparation of the manuscript."

Response: We have added to the financial disclosure "The funders had no role in study design, data collection and analysis, decision to publish, or preparation of the manuscript."

3. Please update your submission to use the PLOS LaTeX template. The template and more information on our requirements for LaTeX submissions can be found at http://journals.plos.org/plosone/s/latex.

Response: We have updated our submission to conform with instructions accessed using links under #1 above.

4. Thank you for stating the following in the Acknowledgments Section of your manuscript:

"The authors acknowledge the contributions of the study participants, site investigators and staff. The study drug was provided at no cost to the Institution by Merck Sharp & Dohme Corp and Gilead Science Inc. The opinions expressed in this paper are those of the authors and do not necessarily represent those of Merck Sharp & Dohme Corp or Gilead Sciences Inc.

The project was funded by NIH R01 DA040532 (ACD). Additional support came from the Molecular Profiling and Computational Biology Core of the University of Washington and Fred Hutch Center for AIDS Research (award number P30 AI027757), from the Laboratory Core of International Maternal Pediatric Adolescent AIDS Clinical Trials Network (IMPAACT) UM1 AI106716 (Subaward: LMF). This work was also supported by the National Center For Advancing Translational Sciences of the National work was also supported by the National Center For Advancing Translational Sciences of the National of the authors and does not necessarily represent the official views of the National Institutes of Health."

We note that you have provided funding information that is currently declared in your Funding Statement. However, funding information should not appear in the Acknowledgments section or other areas of your manuscript. We will only publish funding information present in the Funding Statement section of the online submission form.

Response: Please change the Acknowledgments Section to read: "The authors acknowledge the contributions of the study participants, site investigators and staff; the contribution of the study drugs provided at no cost to the Institution by Gilead Sciences Inc and by Merck Sharp & Dohme Corp~~.~~ The authors acknowledge that the opinions expressed in this paper are those of the authors and do not necessarily represent those of Gilead Sciences Inc or Merck Sharp & Dohme Corp or the official views of the National Institutes of Health."

Please remove any funding-related text from the manuscript and let us know how you would like to update your Funding Statement. Currently, your Funding Statement reads as follows:

"The project was funded by NIH R01 DA040532 (ACD). Additional support came from the Molecular Profiling and Computational Biology Core of the University of Washington and Fred Hutch Center for AIDS Research (award number P30 AI027757), from the Laboratory Core of International Maternal Pediatric Adolescent AIDS Clinical Trials Network (IMPAACT) UM1 AI106716 (Subaward: LMF). This work was also supported by the National Center For Advancing Translational Sciences of the National Institutes

of Health under Award Number KL2 TR002317 (GG). The content is solely the responsibility of the authors and does not necessarily represent the official views of the National Institutes of Health."

Response: Please change the Funding Statement to read: "The project was funded by NIH R01 DA040532 (ACD). Additional support came from the Molecular Profiling and Computational Biology Core of the University of Washington and Fred Hutch Center for AIDS Research (award number P30 AI027757), from the Laboratory Core of International Maternal Pediatric Adolescent AIDS Clinical Trials Network (IMPAACT) UM1 AI106716 (Subaward: LMF) and the National Center for Advancing Translational Sciences of the National Institutes of Health under Award Number KL2 TR002317 (GG). The funders had no role in study design, data collection and analysis, decision to publish, or preparation of the manuscript."

Please include your amended statements within your cover letter; we will change the online submission form on your behalf.

Response: Our amended statements are included within our cover letter.

5. We note that you have indicated that data from this study are available upon request. PLOS only allows data to be available upon request if there are legal or ethical restrictions on sharing data publicly. For more information on unacceptable data access restrictions, please see http://journals.plos.org/plosone/s/data-availability#loc-unacceptable-data-access-restrictions.

In your revised cover letter, please address the following prompts:

a) If there are ethical or legal restrictions on sharing a de-identified data set, please explain them in detail (e.g., data contain potentially sensitive information, data are owned by a third-party organization, etc.) and who has imposed them (e.g., an ethics committee). Please also provide contact information for a data access committee, ethics committee, or other institutional body to which data requests may be sent.

b) If there are no restrictions, please upload the minimal anonymized data set necessary to replicate your study findings as either Supporting Information files or to a stable, public repository and provide us with the relevant URLs, DOIs, or accession numbers. For a list of acceptable repositories, please see http://journals.plos.org/plosone/s/data-availability#loc-recommended-repositories.

We will update your Data Availability statement on your behalf to reflect the information you provide.

Response: There are no ethical or legal restrictions on sharing the de-identified data set. We have added participant de-identified data in spreadsheet labeled "S20 Table" that will allow interested parties to replicate the analyses, as mentioned in our cover letter.

6. Please amend either the title on the online submission form (via Edit Submission) or the title in the manuscript so that they are identical.

Response: We have changed the title in the online submission form to match the manuscript.

7. Please include your full ethics statement in the ‘Methods’ section of your manuscript file. In your statement, please include the full name of the IRB or ethics committee who approved or waived your study, as well as whether or not you obtained informed written or verbal consent. If consent was waived for your study, please include this information in your statement as well.

Response: We have added the full ethics statement and the names of the IRB/Ethics committees to the Methods, as well that we obtained written informed consent.

8. Please include captions for your Supporting Information files at the end of your manuscript, and update any in-text citations to match accordingly. Please see our Supporting Information guidelines for more information: http://journals.plos.org/plosone/s/supporting-information.

Response: We have added captions at the end of our manuscript describing data and updated reference to such in the manuscript body.

[Note: HTML markup is below. Please do not edit.]

Reviewers' comments:

Reviewer's Responses to Questions

Comments to the Author

1. Is the manuscript technically sound, and do the data support the conclusions?

The manuscript must describe a technically sound piece of scientific research with data that supports the conclusions. Experiments must have been conducted rigorously, with appropriate controls, replication, and sample sizes. The conclusions must be drawn appropriately based on the data presented.

Reviewer #1: Yes

Reviewer #2: No

Reviewer #3: Yes

---

2. Has the statistical analysis been performed appropriately and rigorously?

Reviewer #1: Yes

Reviewer #2: I Don't Know

Reviewer #3: Yes

---

3. Have the authors made all data underlying the findings in their manuscript fully available?

The PLOS Data policy requires authors to make all data underlying the findings described in their manuscript fully available without restriction, with rare exception (please refer to the Data Availability Statement in the manuscript PDF file). The data should be provided as part of the manuscript or its supporting information, or deposited to a public repository. For example, in addition to summary statistics, the data points behind means, medians and variance measures should be available. If there are restrictions on publicly sharing data—e.g. participant privacy or use of data from a third party—those must be specified.

Reviewer #1: Yes

Reviewer #2: No

Reviewer #3: No

---

4. Is the manuscript presented in an intelligible fashion and written in standard English?

PLOS ONE does not copyedit accepted manuscripts, so the language in submitted articles must be clear, correct, and unambiguous. Any typographical or grammatical errors should be corrected at revision, so please note any specific errors here.

Reviewer #1: Yes

Reviewer #2: Yes

Reviewer #3: Yes

---

5. Review Comments to the Author

Please use the space provided to explain your answers to the questions above. You may also include additional comments for the author, including concerns about dual publication, research ethics, or publication ethics. (Please upload your review as an attachment if it exceeds 20,000 characters)

Reviewer #1: The present study compared various inflammatory and immune activation biomarkers before HIV acquisition and when participants had a confirmed suppression of viral replication. Several previous studies have evaluated the effects of HIV infection and ART on biomarkers, but the present study provides interesting, previously unpublished information related to those biomarkers. Each participant serves as their own healthy control and that is very interesting and has a great scientific value.
The article is well written, the statistical analysis is well documented and finally results and discussion are clearly presented.

Some minor comments to the authors:
- Pag 8 line 48: could the authors explain why they describe significant intra-participant variability in IP-10, IL-6 and sCD163, when the Holm adjuted p-value is not significant (supplementary table 1)?

Response: We thank the Reviewer for noticing this error, we updated the draft to include both unadjusted and adjusted p-values.

- Figure 2, I suggest modifying “differences” to “change” in ordinate axis. I encourage the authors to include significant p-values in the figure.

Response: We thank the Reviewer for this suggestion, we changed ‘differences’ to ‘log fold change’. In this figure significance is conveyed by the confidence interval. We added this clarification to the legend of Figure 2: ‘This scaling of each analyte to the z scale facilitates presenting them together on a common figure and has no impact on statistical significance, which is communicated here by the CI not crossing the horizontal dotted line at zero. This line reflects no difference between post- and pre-infection values’.

- In all the supplementary tables, I suggest modifying the order of the columns: first the estimated value of the change, second the p-value and third the Holm adjusted p-value.

Response: We thank the Reviewer for this suggestion, we agree and we have changed the order of the columns.

- Supplementary Table 4a: How does the author interpret the difference of significance of p-values between p-value and Holm adjusted p-value?

Response: In S5 Table (previously 4a) both CRP and IFN-α2a showed significant unadjusted p-value but did not sustain significance after Holm adjustment for multiple comparisons. The interpretation is that this paper does not provide primary evidence to support the rejection of the null hypothesis that the ART time initiation has an impact on

the change of any member of the set of measured biomarkers post-infection; however, it raises the possibility that some biomarkers change post-infection in a different way in the deferred versus immediate group. A larger dataset will have to be analyzed to further evaluate whether these biomarkers systematically differ across PWH depending on timing of ART initiation.

- Page 9 line 191. I think it should be the horizontal dotted line.

Response: We thank the Reviewer for noting this error, and we have changed the legend for Figure 2 to refer to the horizontal dotted line.

Reviewer #2: "Article: Observational Study of the Effects of HIV Acquisition and Antiretroviral Treatment on Biomarkers of Systemic Immune Activation"

In this article, the authors hypothesized that (1) individuals living with HIV (PWH) would demonstrate greater immune activation after ART suppression of viral replication compared to pre-infection values, and (2) PWH initiating ART at the time of diagnosis during acute or early primary infection (referred to as "immediate" ART) would show less immune activation compared to those who deferred ART for approximately 24 weeks.

The authors concluded that certain plasma biomarkers, including CPR, IP-10, MCP-1, and TNFa, were activated, while leptin was inactivated after HIV infection with viral suspension. These results were consistent with previous reports.

As mentioned by the authors, this study is unique as it documents changes in biomarkers of immune activation in individuals with prospectively documented incident HIV infection and examines differences in biomarkers between participants randomized to initiate ART immediately in early infection or to defer ART for 24 weeks. However, some parts of the article, especially the graphs and calculations, are confusing.

Here are the comments:

Major comments

On page 6, lines 129 to page 7, line 135, the description of "Time interval from EDDI to ART in days" in the text and Table 1 is confusing. The reviewer understands that Table 1 describes the "as-treated analysis". Please include the actual numbers for "immediate-ART" and "defer-ART" in the table. Additionally, clarify in the "Method" where the 3 participants who initiated ART between "immediate" and "defer" were analyzed.

Response: We thank the Reviewer for noting that the "Time interval from EDDI to ART in days" in the text and Table 1 is confusing. We have modified the Table to include the

actual numbers for "immediate-ART" and "deferred-ART", as well as numbers for both the as-treated and per-protocol assignment. Additionally, we have modified the "Participants " section in the Results, to clarify how the 3 participants who initiated ART between "immediate" and "deferred" were analyzed. Finally, we reviewed and ensured that all analyses are presented with the number of samples (N).

The reviewer recommends revising Figure 1 to be a "violin plot" or "boxplot" to better display the distribution of the biomarkers, as it is currently unclear. The authors concluded that CRP, LBP, IP-10, leptin, and TNF-a were significantly different between the "pre-" and "post-" HIV infection stages. However, the plot appears to include each other. Also, in the description of Figure 1, the authors mentioned that "The mean biomarker levels of most participants remained within established norms (page 8, line 154). However, some biomarkers such as suPAR, CRP, IL-6, leptin, and TNF-a appear higher than the normal level. Do the authors have any insight into whether the MSM population shows higher values for these biomarkers?

Response: We revised Figure 1 to convey our findings more clearly: we changed dot plot to swarm plot, removed p-values as this information is redundant with Figure 2 and S4 Table; we also highlighted the cases with values above the normal range.

We agree with the Reviewer that a comparison of pro-inflammatory biomarkers between MSM populations with and without HIV infection would be of interest to help us understand whether factors in addition to HIV are leading to relatively higher values of pro-inflammatory biomarkers in MSM compared to heterosexual men. Unfortunately, we could not find data comparing these populations. In the population we studied, ethanol was frequently used by participants which may have contributed to increased pro-inflammatory biomarkers prior to HIV infection.

Additionally, does CRP need a symbol for "Holm adjusted p-value <0.001"?

Response: Yes, thank you for detecting this error. We have revised Figure 1 as suggested to better display the distribution of the biomarkers. We decided to remove from this figure p-values as this information is redundant with Figure 2 and S4 Table. Instead, we decided to focus on showing our data in the context of normal ranges.

The Y-axis title "Difference between Post- and Pre-infection in SD units" of Figure 2 is confusing. Please explain why the "difference" needs to be "normalized by SD". The reviewer believes that the unit of Figure 2 should be portrayed as a "fold difference". For example, does CRP elevate by ~35% after infection? Please confirm.

Response: We thank the Reviewer for the suggestion, we changed ‘differences’ to ‘log fold change’. We normalized our data by SD to display multiple biomarkers on a common scale. For CRP ~0.35 means that post-infection $\log_{10}$ CRP levels increased the number of times equal to ~35% of the pre-infection SD for $\log_{10}$ CRP. The non-

normalized data can be found in the S4 Table. We also ensured that the description of supplementary tables contains a sentence describing the interpretation of the results, as well as Figure 2: “Estimates and confidence intervals can be interpreted as a $\log_{10}$ fold-change difference between the two conditions expressed in units of the pre-infection standard deviation of a given analyte (expected fold-change pre-challenge)”.

In the "Conclusion", the authors suddenly mentioned "Given the strong association of CRP with cardiovascular disease these findings emphasize that HIV prevention and ART initiation during primary infection could diminish non-AIDS events", however, there is no further comments for this, such as this CRP fold change might be read a risk factor for cardiovascular disease. Please comment for this. In addition, what the authors are thinking an application for elevation of IP-10, MCP-1, and TNFa, and also depression of leptin? Or do authors thing these change are clinically relevant?

Response: Thanks for pointing out that the Conclusion seems to suddenly link findings to clinical outcomes. The conclusions have been revised to explain in more detail our interpretation of the relevance of our findings to understanding the mediators of systemic inflammation and potentially the clinical outcomes in people living with HIV.

Minor comments

Page 5, line 87: Did the authors confirm non-HIV infection for Visit 2 samples?

Response: Yes, all 50 participants had a testing at a visit subsequent to Visit 2 and in 48 this visit produced results and was at a time that confirms that they were HIV uninfected at Visit 2. The results from two do not clearly indicate the timing of HIV infection.

Specifically, following Visit 2, 43 of 50 participants tested HIV RNA negative and seronegative at a subsequent visit; five were HIV RNA positive but seronegative at a visit >6 weeks after Visit 2; and the two remaining participants (who were HIV RNA positive and seronegative at Visit 2) were HIV RNA positive and seropositive at their next visit 8-12 weeks following Visit 2. There is a remote chance that these last 2 had acute HIV infection at Visit 2, but the remainder were clearly HIV-uninfected at Visit 2.

Page 5, lines 97-100: We understand that there is a large inter-analytical variation for biomarker ELIZA analysis. What is the variation of each analyte (was it an average of multiple analyses) for each biomarker? And did this variation affect the final data analyses?

Response: All four of each participant’s specimens were assayed in a single ELISA run to avoid inter-analytical variation.

Page 6, lines 120-122: Please explain the utility of "mean/SD" for data analysis. The reviewer believes that "mean/SD" is an indicator of variation, and when it is small, the data variation is also small. However, the authors used it to normalize the magnitude. Please explain the validity of this approach.

Response: The mean/SD was not used for data analysis, only for data visualization. To display multiple biomarkers on a common scale we took the difference between pre- versus post-infection means and divided them by the pre-infection standard deviation for each biomarker. This also aids in interpretation since the results are displayed in terms relative to the expected variation during the pre-infection time period. The text has been modified to explain our approach to data analyses and display.

Supplemental Table 3: The reviewer believes that the difference between "as-treated" and "per-treated" involves approximately 5 participants, yet some of the biomarkers' estimated fold changes were large. Did the data from these participants highly influence the results? Please provide an explanation. With regard to the "estimated change," does it refer to the fold change of "post-dose" compared with "pre-dose", even if the number was normalized by SD? For example, the estimate of CRP is 0.21, indicating a 21% higher value for the "post-treatment."

Response:

The difference between ‘as-treated’ and ‘per-protocol’ involves 5 participants. In the ‘per-protocol’ analysis these five are in the deferred-ART group, in the ‘as-treated’ analysis 2 of these five, who initiated ART within the timeframe of the immediate group are included in the immediate-ART group for analyses and 3 of these five, who initiated ART between timeframes of immediate and deferred are excluded. S6, S7, S16 and S18 Tables show details of within-treatment-group estimates for the treatment groups for “per-protocol” and “as-treated” analyses. While some estimates change across these analyses, as shown in S5 and S14 Tables, the findings of which biomarkers significantly differed across treatment arms were not sensitive to these different analyses sets. With regard to the "estimated change," in S4 and S12 Tables , formerly 3a and 3b: it refers to the $\log_{10}$ fold change of ‘post-infection’ compared with ‘pre-infection’, the number was not normalized by SD. The estimate of CRP being 0.21 indicates that the ‘post-infection’ $\log_{10}$ CRP mean value is the pre-infection $\log_{10}$ CRP mean value plus 0.21, and that on the originally measured scale, the geometric mean CRP value is 10^0.21 (1.62) times greater post infection than pre-infection. Normalization by SD was only used for data visualization and to ease interpretation. It has no effect on the statistical analysis as it is a constant scalar. We also ensured that the description of supplementary tables contains a sentence describing the interpretation of the results: “Estimates and confidence intervals are expressed as a difference in means on the $\log_{10}$ scale and can be interpreted as a log10 fold-change of geometric means between the biomarker values measured in the two conditions.”

Reviewer #3: In the article ‘Observational study of effects of HIV Acquisition and Antiretroviral Treatment 1 on Biomarkers of Systemic Immune Activation’ submitted for review at PLOS One, the authors use a retrospective study comparing inflammatory biomarker measurements collected before and after ≥2 years of effective ART in men-who-have-sex-with-men (MSM) and transgender women in Lima, Peru. The article is well written, easy to follow, and the conclusions seem generally plausible given the results of the statistical analyses. However, I do have a few comments:

Major Comments

1. Page 6, lines 115-116: ‘This effectively allowed us to implement a pooled variance one-sample t-test with repeated measures, treating the paired visits within the pre- or post-infection timeframe as exchangeable.’
- I do have some concerns about the analysis design. Firstly, I do think this part can be strengthened with a better explanation. It is not clear how the paired t-test is conducted between the pre- and post- ART timepoints from this explanation, but looking at Figure 1, it seems that the participant-specific paired values for the t-test are the average of their two pre- ART timepoints and the average of their two post- ART measurement. Can the authors please elaborate more on this in the draft?

Response: Thank you for pointing out that our description is insufficient. We added the following sentence: ‘The participant-specific paired values for the t-test are the average of their two pre-infection timepoints and the average of their two post-infection measurements. In addition, we performed a robustness analysis for the unstable biomarkers in which we used Visit 2 to represent a pre-infection measurement and Visit 4 to represent a post-infection measurement.

Also, with four distinct biomarker measurements, I am curious as to why the authors decide to conduct a pre-post design by pooling the pre- and post- infection timepoints together.

Response: Our intention in selecting these samples was to increase precision for comparing a pre-infection to a post-infection steady state. Alternative designs and additional cohorts with finer-grained post-acquisition sampling may be better suited to investigating patterns of transient changes, or of long-term trends in biomarkers post acquisition.

Also, the assumption of exchangeability seems to have failed for some of these biomarkers (Supplementary Figures 1 and 2), given that the difference of the two pre- and post- ART measurements are statistically different than 0 for them. In that light, I

am wondering why multiple measurement models, like the mixed-effects models or GEE were not pursued here.

Response: In our view these data are not well suited to more complex models, given that simpler models fit well for the most part, and given other limitations of this design. We agree that the finding of non-exchangeability might justify employing more complex models for the subset of “unstable” markers, such as models that employ numerical elapsed time rather than ordinal visit numbers, and (as suggested by the reviewer) models that account for more complex variance and covariance structures. In our exploratory analyses of these data, we did not find that this data set is well suited for that kind of analysis. In our view there is not enough evidence to justify the imprecision of fitting complex variance models here, and while we know many practitioners would employ a mixed-effects model or GEE in that circumstance, we come from an Occam’s Razor school of applied statistics, which includes a resistance to introducing untestable distributional assumptions when a simpler approach can directly address the scientific question. In our view, the question of changes from pre-to-post infection and difference in these by ART start time are better addressed by focusing on two relevant time points (visits 4 and 2) and providing interpretable and robust statistical analyses of them as we have done in our robustness analysis here, than by introducing statistical model-based accommodation for the violated exchangeability assumptions of the primary analysis. For future work, more longitudinal data post-infection could be used to identify time trends; this would plausibly require a curve registration step for the post-infection data because even in calendar elapsed time, not every longitudinal pattern of marker changes post infection will align across participants in time since diagnosis or in time since EDDI. With only two post-infection sample times per participant with a large inter-sample spacing and with relatively low consistency across participants in the elapsed times of these samples since diagnosis, there is little evidence in these data to select from possible alternative modeling approaches, and little data to fit a complex model of either the mean or the variance structure. Our approach here is to conduct an interpretable analysis of the available data. In a previous paper, we investigated a larger longitudinal cohort (of which the 50 participants in this manuscript are a selected subset) to identify pre-infection biomarkers associated with HIV acquisition (PMID: PMC9722478). Further work to evaluate marker levels on longitudinal post-infection samples in this or another cohort could help to elucidate patterns of consistent marker level changes such as durations of transient effects of HIV acquisition and ART treatment.

Also, it may make more sense to pool the two pre-ART timepoints, but given the two post- ART timepoints are 18 months apart, and given that timing of ART and the course of ART over time may have a temporal impact on expression of these biomarkers, what is the plausibility of pooling the two post- ART timepoints together?

Response: Our finding, as we show in S3 Table, is that it is generally plausible to pool them insofar as we do not find significant evidence of post-acquisition trends that is consistent across participants, but as we show there are individual markers for which we do have evidence of a difference sufficient to reject that null hypothesis. The data support that our lack of finding such trends in the other markers is not simply a low power problem. We understand the reviewer’s perspective that a priori we might not

assume post-acquisition levels achieve stability by the time of our first post-acquisition evaluation, or perhaps ever. Due to particulars of this data set, it is relatively well suited to treating the post-acquisition time points as two measures of the same longitudinal steady state. While pre-infection samples at Visit 2 are well-registered across participants relative to diagnosis date and EDDI, other samples are spaced sporadically over time (see S20 Table). An alternative design in a future study, with well-registered close-to-diagnosis post-acquisition longitudinal samples spaced close together might be better suited to evaluating transient effects.

Minor Comments

1. Page 5, lines 86-88: 'Specimens included one plasma sample shortly after enrollment, a second ≤3 months from EDDI (Visits 1 and 2, respectively)': The second sample was ≤3 months before or after the EDDI? In case it is after, can it be confirmed that it is still a pre-ART timepoint?

Response: All Visit 2 samples were prior to ART initiation (see Response to Page 5, line 87, above).

2. How is the 'per-protocol' analysis defined (as reported in Supplementary Tables 3b, 4b, 5b, and 6b). I don't think it was mentioned anywhere in the draft.

Response: We have modified the draft to clarify how the 'pre-protocol' analysis was defined: "From among a total of 216 Sabes participants with prospectively documented incident HIV infection 50 had specimens from four time-points and fulfilled ART-suppression entry criteria and were used for this study of immune biomarkers. These 50 included 19 participants randomized to immediate-ART and 31 randomized to deferred-ART. Data from all 50 participants were used in assessing the biomarkers' stability and the "per-protocol" analysis. "

3. One requirement for the data availability statement is that 'data available on request from the author' is not a sufficient response, and if data are indeed only available upon request, the authors must answer 'No' for the question - 'Do the authors confirm that all data underlying the findings described in their manuscript are fully available without restriction?' In the manuscript information page (Page 4), the authors answered 'Yes' to the aforementioned question, but state in data availability statement that 'Summary data available in the manuscript. Participant level data is stored at Fred Hutch and de-identified data is available by request of Ann Duerr'. This is a contradiction that they need to resolve.

Response: As mentioned above in response to the Editor, there are no ethical or legal restrictions on sharing the de-identified data set. We have added S20 Table with de-identified data that will allow interested parties to replicate the analyses, as mentioned in our cover letter.

4. Given the number of biomarkers assessed, multiple testing adjustment is indeed necessary to parse out the results, and in this regard, I do appreciate that the authors have put careful thought into this and presented Holm adjusted p-values with their results. In the description of results in Page 9 (lines 173-182), it is important to point out both sets of results. For example, the authors start by stating,
'Comparisons of all participant's (N=47) mean pre-infection biomarker values to their ART-suppressed mean values by a regression analysis detected statistically significant increases in IP-10, MCP-1/CCL2, TNFα, CRP and significant decreases in leptin and LBP (Figure 1), with differences sustained after Holm adjustment for multiple comparisons in all but LBP and TNF-α (Supplementary Table 4a).'
This is precisely how these results should be presented in my opinion (with conclusion from the unadjusted and adjusted analyses). However, in the next set of sentences (lines 172-183), only results from the unadjusted analyses are given. Can the authors please add the results from the adjusted analyses as well, as has been done in the first sentence of that paragraph.

Response: Thanks for pointing out this inconsistency. We added the adjusted analyses to all biomarkers that are discussed in the revised manuscript.

---

6. PLOS authors have the option to publish the peer review history of their article (what does this mean?). If published, this will include your full peer review and any attached files.

If you choose "no", your identity will remain anonymous but your review may still be made public.

Do you want your identity to be public for this peer review? For information about this choice, including consent withdrawal, please see our Privacy Policy.

Reviewer #1: No

Reviewer #2: No

Reviewer #3: No
